# Supplementary material for: Exploring the socioeconomic determinants of dietary diversity in rural Bangladesh: A longitudinal study
Source: PLoS One. 2025 Feb 27;20(2):e0317623. doi: 10.1371/journal.pone.0317623 (PMC11867316; doi:10.1371/journal.pone.0317623)
Supplement: Attachment — Submitted filename: response-to-reviewers.pdf [file pone.0317623.s001.pdf]

PONE-D-24-47837
Response to Reviewers

Dear Editor,

We appreciate the thoughtful feedback provided by the reviewers and the opportunity to improve our manuscript titled *"Exploring the socioeconomic determinants of dietary diversity in rural Bangladesh: A longitudinal study"*. Below, we address each comment raised by the reviewers, providing detailed responses and outlining the changes made in the manuscript.

Reviewer 1 Comments

Comment 1: *Figures and tables: Review Table 4 and I would start with this table (demographics) then move to Table 3 – just a suggestion.*

Response: Thank you for the suggestion. We have revised the order of the tables and now present Table 4 (demographics) before Table 3, which we believe improves the logical flow of the results.

Comment 2: *Study design: A little more detail to the construction of the balanced panel dataset to allow for replicability of arriving at the 4,593 per year (lines 71-81).*

Response: We have added more details on the construction of the balanced panel dataset specifying the steps and criteria used to arrive at 4,593 observations per year (lines 88–94).

Comment 3: *Results and data: The results are presented very well though it would be nice to have the results cover the demographics first (lines 178-190).*

Response: We have reorganized the results section to present the demographics first, as suggested, to improve the flow of the presentation.

Comment 4: *Results and data: The female headship increased – in the discussion this should be pointed out for potential follow-up studies (line 201).*

Response: We have highlighted the increase in female headship in the discussion section and suggested it as an avenue for future research (line 236–240).

Comment 5: *Results and data: Gender of household head – it would be nice to have a discussion around this result too (lines 243–244).*

Response: Thanks for the suggestion. We have added a discussion on the gender of the household head and its implications (lines 379–382).

Comment 6: *Results and data: The paper might need to describe what "Larger households" means in their context (line 296).*

Response: Thank you for pointing this out. By "larger households", we simply referred to households with more members compared to others with fewer members; no specific cut-off point was established. To avoid further confusion, we have revised the sentence as follows:

"The number of household members is positively associated with dietary diversity, as households with more members often benefit from shared resources and collective purchasing power." (line 384–386)

Comment 7: *Results and data: Limitations of the methods could also mention working with secondary data (line 307).*

Response: We have included a note on the limitation of using secondary data on line 463–464.

Comment 8: *Conclusion: The conclusion could be improved (lines 327-336).*

Response: We have revised the conclusion section, and it now provides a stronger summary of the findings and implications (lines 485–509).

Comment 9: *References: 1996 reference might be a little old, is it possible to use a more recent citation? (lines 2-3).*

Response: We have updated the 1996 reference to a more recent citation (lines 2–4).

Reviewer 2 Comments

Comment 1: *Abstract: While the abstract is well-structured, it could benefit from greater specificity regarding the magnitude of the effects of key variables and a clearer explanation of the theoretical framework guiding the study. Also, placing the findings within a broader global context, particularly in relation to the Sustainable Development Goals (SDGs) on food security, would further highlight the significance of the study's contributions to both academic and policy discussions on improving dietary diversity and food security in rural communities.*

Response: Thank you for the suggestion. We have revised the abstract to include specific details on the magnitude of the effects of key socioeconomic variables to enhance the reader's understanding. Additionally, we have contextualized our findings within the broader scope of the Sustainable Development Goals (SDGs), particularly Goal 2 (Zero Hunger), and Goal 3 (Health and Well-being).

Introduction:

Comment 2: *However, while the introduction highlights significant socioeconomic determinants of dietary diversity, it could benefit from a clearer identification of specific research gaps in rural Bangladesh, particularly with respect to factors such as gender, access to healthcare, and regional disparities.*

Response: We have revised the introduction and highlighted key research gaps in rural Bangladesh, focusing on gender, occupation, education of the household head, expenditure, and resource access (lines 36–39).

Comment 3: *The introduction also introduces the Poisson regression model as a methodological approach but does not provide sufficient detail on why this model is particularly appropriate for the study, which could enhance the reader's understanding of the analytical rigor.*

Response: Thank you for the comment. We have provided an explanation in the revised introduction (lines 51–56) on why Poisson regression is particularly suitable for this study.

Comment 4: *Additionally, while the study mentions the use of panel data, a more explicit discussion of how this approach contributes to the robustness of the findings would be valuable.*

Response: We have added a short discussion on how the panel data approach improves the robustness of our findings (lines 46–50).

Data, Sampling and Variables

Comment 5: *However, while the study covers many critical factors, it could benefit from a deeper focus on gender, especially in terms of intra-household food distribution and decision-making, as gender dynamics often play a significant role in food security.*

Response: Thanks for the suggestion. This study particularly examines dietary diversity at the household level rather than the individual level. Accordingly, gender is analyzed in the context of the household, specifically through the gender of the household head. This approach aligns with the study's household-level focus and enables an assessment of the broader influence of gender on household dietary diversity. The manuscript has been updated for clarification (lines 135–138).

Comment 6: *The operationalization of HDDS and FVS is generally clear, though excluding food consumed outside the home in the HDDS calculation may limit the comprehensiveness of dietary diversity assessment.*

Response: Thanks for the comment. The HDDS indicator is specifically designed and validated to measure household-level access to food, rather than individual-level

access [1]. In rural, low-income settings, household members typically consume meals prepared at home, even when working away during the day. As a result, food purchased and consumed outside the home is uncommon. However, we acknowledge that this exclusion may still limit the comprehensiveness of dietary. We have noted this as a potential limitation in the manuscript (line 450). The manuscript has been also updated for clarification (lines 112–118).

Comment 7: *Additionally, variables such as access to markets, local agricultural policies, or seasonal variations in food availability are not considered, which could be significant in rural settings.*

Response: Thank you for the comment. These variables are not available in the secondary data we used. Access to markets is only available for farming households, which is why it couldn't be included in our balanced panel.

Exploratory Analysis

Comment 8: *However, while the methodology is robust, some potential limitations need consideration. First, while the fixed-effects model controls for unobserved household-specific heterogeneity, it may not fully address temporal effects or interactions between unobserved variables over time. The study also assumes linear relationships, which may oversimplify the dynamics of dietary diversity. Further, the study could benefit from exploring alternative models to handle potential endogeneity or selection bias, particularly given the socio-economic variables involved.*

Response: Thank you for this insightful comment. We agree that advanced modeling approaches, such as those addressing potential endogeneity and selection bias, could further enhance the robustness of the analysis. However, implementing such approaches is beyond the scope of this study, which focuses on employing fixed-effects models to control for household-specific unobserved heterogeneity. We acknowledge this as a limitation of our study and have noted it as an area for future research to explore more comprehensive methodologies. (lines 460–463)

Results

Comment 9: *This methodological approach ensures that unobserved heterogeneity is accounted for, enhancing the reliability of the study's findings. However, I recommend expanding the rationale behind the choice of Poisson regression, particularly for a broader audience, by providing a more detailed explanation of its advantages and why it is the most suitable method for this type of analysis.*

Response: We revised the manuscript to expand the rationale behind the Poisson regression for a broader audience (lines 180–186).

Comment 10: *The manuscript also examines several socioeconomic factors influencing dietary diversity, but there is a need for a more in-depth exploration of why certain variables, such as marital status and negative shocks, show inconsistent associations across the two dietary diversity indices (HDDS and FVS). A deeper understanding of these inconsistencies could provide valuable insights into how these factors interact with other variables or influence dietary diversity in different contexts.*

Additionally, the findings related to religion, specifically the potential influence of cultural or dietary practices among Muslim households, should be further contextualized. Given the significant role that religion can play in shaping dietary habits, especially in rural settings, it would be beneficial to discuss how specific practices, such as fasting or dietary restrictions, may impact dietary diversity in Muslim households.

Response: Thanks for the suggestion. We have revised the manuscript and added a subsection, "Negative shock, Religion, Marital status, and Time" in the Discussion section that discusses the effect of marital status, negative shocks, and religion on dietary diversity (lines 408–431).

Comment 11: *The manuscript would also benefit from a more detailed discussion of potential confounders or omitted variables, such as income or wealth, which could influence the results. The omission of these factors could bias the findings and affect the interpretation of the study's implications for policy. Including a consideration of these variables would enhance the robustness of the analysis and provide a more nuanced understanding of the socioeconomic determinants of dietary diversity.*

Response: Thank you for the comment. We have used household food consumption expenditure per capita and non-food consumption expenditure per capita as proxies for income, given the lack of direct income data. While total wealth is not included as a covariate, we account for wealth through proxies like land and home ownership, which reflect access to productive assets and financial stability in rural settings.

We have revised the manuscript for clarity (lines 144–159)

Comment 12: *Lastly, while the manuscript reports improvements in dietary diversity over time, it would be helpful to clarify how the survey rounds themselves may have influenced these results. For instance, changes in household circumstances, regional interventions, or broader socio-economic shifts could contribute to the observed improvements. A discussion on how these temporal factors interact with the study's findings would strengthen the manuscript and provide additional context for interpreting the changes in dietary diversity across the survey rounds.*

Response: Thank you for the comment. We have revised the manuscript and discussed the impact of survey rounds on dietary diversity and its implications (lines 432–441)

Discussions

Comment 13: *Addressing Time-Invariant Factors: While the use of a fixed-effects model is appropriate for controlling unobserved heterogeneity, the inability of this model to account for time-invariant factors, such as geographical location, is a limitation. The authors briefly mention this, but further discussion on how these unmeasured factors may influence dietary diversity would enhance the interpretation of the results. Exploring alternative methodologies, such as random-effects models, could also provide additional insights into the role of these factors.*

Response: Thank you for the feedback. We acknowledge that while the fixed-effects model controls for unobserved household-specific heterogeneity, it is unable to account for time-invariant factors, such as geographical location. To address this, we have added a "Robustness Checks," section in the Appendix, which explores alternative models, including random effects, non-linear relationships, and models with interactions. (Appendix A)

Comment 14: *Mechanisms Behind Education's Role: The paper identifies education, particularly primary education, as a key determinant of dietary diversity. However, the study could benefit from a more detailed exploration of the mechanisms through which education influences dietary practices. A deeper discussion of how education impacts food choices, such as through increased nutritional awareness or improved economic decision-making, would provide a clearer understanding of the pathways linking education to dietary diversity.*

Response: Thanks for the comment. For a deeper discussion on how education impacts food choices, we have revised the manuscript and created a separate subsection, "The Role of Education" in the Discussion Section. (lines 341–359).

Comment 15: *Farming and Dietary Diversity: The relationship between farming as the primary occupation and greater dietary diversity is intriguing but requires further exploration. It would be helpful to investigate the underlying mechanisms driving this association. For example, is the increased dietary diversity a result of farmers having access to a wider variety of food sources, or is it due to higher income levels that enable more diverse food purchases? A more detailed analysis of agricultural practices and economic outcomes would help clarify these pathways.*

Response: Thanks for the comment. To investigate the underlying mechanism driving the relationship between farming and dietary diversity, we have revised the manuscript and created a separate subsection, "The Impact of Occupation and Agriculture," in the Discussion Section (lines 361–377).

Comment 16: *Expenditure Patterns: The study identifies food and non-food expenditures as important determinants of dietary diversity, but further discussion is needed on how these expenditures translate into food choices. Are higher food expenditures associated with*

a preference for more expensive, diverse foods, or do households prioritize bulk purchases of cheaper, less diverse options? Understanding how households allocate their financial resources in the context of dietary choices would enhance the depth of the analysis.

Response: Thank you for the comment; we have revised the manuscript and expanded the discussion on how food and non-food expenditures influence dietary choices (lines 387–397).

Comment 17: *Methodological Transparency: While the authors mention the use of 7-day recall data, a more thorough explanation of why this method was chosen over the 24-hour recall would improve the clarity and transparency of the methodology. Also, addressing potential biases associated with recall-based dietary assessments, particularly in rural settings where memory recall may be less reliable, would strengthen the validity of the results.*

Response: Although we had already mentioned that using a 7-day recall is a limitation of the study, we revised the manuscript and added a more thorough explanation, and addressed the potential memory recall bias (lines 446–452).

Comment 18: *Interactions Between Determinants: The study identifies several socioeconomic determinants of dietary diversity, but it would benefit from a deeper analysis of how these factors interact with each other. For instance, how does the education level of the household head interact with occupation or household size in influencing dietary diversity? Understanding these interactions could provide a more comprehensive picture of the complex dynamics influencing dietary diversity.*

Response: Thank you for your comment. We explored several potential two-way interactions, but all were found to be insignificant in the fixed-effects model. This is discussed in the "Robustness Checks" section of Appendix.

Conclusions

Comment 19: *However, the conclusion could be strengthened by further elaborating on the underlying mechanisms that link the identified determinants to dietary diversity. Specifically, a more detailed discussion of how education, agricultural practices, and financial resources influence dietary choices would provide a deeper understanding of the dynamics at play.*

Response: We revised the conclusion accordingly (lines 489–496).

Comment 20: *Additionally, the conclusion would benefit from more explicit consideration of time-invariant factors, such as geographical location, and how these may influence the results.*

Response: Thank you for the suggestion. Since the Hausman test indicated that the fixed-effects model is appropriate for the data, our analysis primarily focuses on time-variant factors. Nevertheless, the "Robustness Checks" section in the Appendix discusses the influence of the time-invariant factor, geographical location, on dietary diversity. Given that Hausman test supports the fixed-effects model for this study, the results from a random-effects model may not be reliable. Therefore, we decided to not include random-effects results in the conclusion.

Comment 21: *A deeper exploration of the role of financial constraints, particularly in terms of how households allocate their resources, would also enhance the study's practical applicability.*

Response: We revised the conclusion accordingly (lines 485–491).

Thank you again for your valuable feedback. We believe these revisions have strengthened our manuscript significantly.

References

1. Swindale A, Bilinsky P. Household Dietary Diversity Score (HDDS) for Measurement of Household Food Access: Indicator Guide (v.2). Washington, DC: FHI 360/FANTA. 2006;.
